# Potential Use of Antiviral Agents in Polio Eradication

**DOI:** 10.3201/eid1404.070439

**Published:** 2008-04

**Authors:** Armando M. De Palma, Gerhard Pürstinger, Eva Wimmer, Amy K. Patick, Koen Andries, Bart Rombaut, Erik De Clercq, Johan Neyts

**Affiliations:** * University of Leuven, Leuven, Belgium; † University of Innsbruck, Innsbruck, Austria; ‡ Pfizer Global Research and Development, San Diego, California, USA; § J&J Pharmaceutical Research and Development, Beerse, Belgium; ¶ Vrije Universiteit Brussel, Brussels, Belgium

**Keywords:** Poliovirus, Poliomyelitis, Antiviral agents, synopsis

## Abstract

In 1988, the World Health Assembly launched the Global Polio Eradication Initiative, which aimed to use large-scale vaccination with the oral vaccine to eradicate polio worldwide by the year 2000. Although important progress has been made, polio remains endemic in several countries. Also, the current control measures will likely be inadequate to deal with problems that may arise in the postpolio era. A panel convoked by the National Research Council concluded that the use of antiviral drugs may be essential in the polio eradication strategy. We here report on a comparative study of the antipoliovirus activity of a selection of molecules that have previously been reported to be inhibitors of picornavirus replication and discuss their potential use, alone or in combination, for the treatment or prophylaxis of poliovirus infection.

## Abstract 2

These compounds may serve as starting points for the design of more potent poliovirus inhibitors.

The Global Polio Eradication Initiative (GPEI) was launched by the World Health Assembly 20 years ago. The principal idea behind the GPEI was to eliminate polio worldwide by the year 2000 by means of large-scale vaccination with the oral live attenuated polio vaccine (OPV) developed by Albert Sabin (*1*). The GPEI has resulted, since 1988, in a decrease in poliomyelitis cases from 350,000 to <2,000 (*2*,*3*). Today, poliovirus (PV) is endemic in 4 countries (Nigeria, India, Pakistan, and Afghanistan), whereas the virus was prevalent in >125 countries at the time the initiative was launched (*4*). When wild PV transmission has been interrupted, the World Health Organization proposes ending the global routine OPV to prevent the risk for vaccine-associated paralytic poliomyelitis, chronic infection of immunodeficient persons, and the reestablishment of poliomyelitis through circulating vaccine-derived PV (*5*). A panel was convened by the National Research Council to evaluate the potential for an antiviral drug as one of the tools to minimize poliomyelitis risk after OPV cessation. The conclusion of the panel was that it would be appropriate, and possibly essential, to develop antiviral drugs for PV infection, as an additional tool to address the problems that might arise in the “postpolio” era (*6*). Antiviral agents do not confer immunity but could be used prophylactically as well as therapeutically. They could protect inactivated polio vaccine (IPV) recipients from PV infection, limit spread until immunity can be ensured and help clear vaccine-derived PV from persistently infected persons (*7*). The ideal drug would be safe, inexpensive, easy to use, stable, and manifest broad activity toward PV strains.

To date, few, if any, drug discovery programs for PV have been initiated. Therefore, research initiatives leading to the successful development of anti-PV drugs will have to rely on the current knowledge of existing picornavirus antiviral agents. Antipicornavirus compounds that reached clinical trials are scarce, and despite the fact that some of these drugs have demonstrated activity against certain picornavirus-associated conditions in humans, no specific antipicornavirus agent has yet been approved by the US Food and Drug Administration (FDA) (*8*).

A substantial number of small molecule compounds have been reported as potent inhibitors of the replication of picornaviruses in vitro (*8*). These compounds could serve as scaffolds for the development of more potent and selective inhibitors of PV. The information available on their structure-activity relationship and their mechanism of action could be exploited as a solid base for developing a specific anti-PV therapy.

We report on a comparative study of a selected series of antipicornavirus drugs for their ability to inhibit PV replication in vitro. The unique aspect of this report lies in the fact that 1) certain drugs (e.g., rupintrivir) were specifically developed to treat rhinovirus and other infections and have never been evaluated for their ability to block PV replication and 2) the selected compounds have never been compared in parallel by using the same technique against the 3 vaccine strains.

## Rationale for Selection of Antipicornavirus Drugs

**Figure 1:**
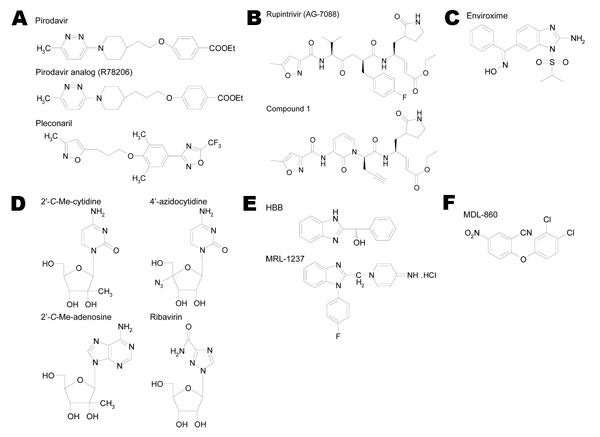
Structural formulas of selected poliovirus inhibitors. A) Capscid binders; B) protease inhibitors; C) 3A inhibitor; D) nucleoside analogs; E) 2C inhibitors; F) unknown target. HBB, 2-(α-hydroxybenzyl)-benzimidazole.

Because this study was triggered by the recognition that antiviral drugs will be needed in the postvaccination era as a countermeasure against the persistence or reemergence in the environment of vaccine-associated virus, we decided to confine our study to the 3 Sabin strains used for vaccination. The aim was to include compounds that act on different targets in the picornavirus replication cycle (preferably 1 or 2 compounds per target) (Figure 1). When a rather large number of molecules had been described that act through the same target (e.g., for the capsid binding agents), we selected those compounds that were in the most advanced state of development and preferably had been studied in a clinical setting. When only 1 or a few compounds had been described for a particular target (for example, with enviroxime, the sole protein 3A–targeting drug reported so far), the impact in the clinical setting was considered less important. Ribavirin was included as a reference standard, since it was regarded as a broad-spectrum inhibitor of positive-strand RNA viruses.

## Methods

The antiviral and cytotoxic activities of the selected compounds were initially determined by means of a cell protection assay. In this assay, a soluble tetrazolium compound (3-[4,5-dimethylthiazol-2-yl]-5-[3-carboxymethoxyphenyl]-2-[4-sulfophenyl]-2H-tetrazolium [MTS]), when used in combination with an electron transfer reagent (phenazine methosulfate [PMS]) is bioreduced by viable cells in culture, a reaction that is colorimetrically quantified. The antiviral and cytotoxic activities were expressed as mean effective concentration (EC_50_, the compound concentration that inhibits virus-induced cytopathic effect [CPE] formation by 50%) and 50% cytotoxic concentration (CC_50_). Therapeutic indexes (TIs) were expressed as the ratio between the CC_50_ and the EC_50_. Briefly, HeLa cells (ATCC CCL-2), grown to confluency in 96-well plates, were infected with 100 50% cell culture infectious doses of virus. After an adsorption period of 2 hours at 37°C, unadsorbed virus was removed and serial dilutions of the compounds were added. The cultures were further incubated at 37°C for 3 days, until complete CPE was observed in the infected and untreated virus control. For cytotoxicity determination, uninfected cultures were incubated with dilution series of compound for 3 days at 37°C. After removal of the medium, 90 µL medium and 10 µL MTS/PMS (Promega, Leiden, the Netherlands) were added to each well; after an incubation period of 2 hours at 37°C, the optical density of each well was read at 498 nm in a microplate reader. EC_50_ values were calculated as previously described (*9*).

## Results

### Capsid-binding Agents

One of the best studied targets for antiviral therapy in picornaviruses is a hydrophobic pocket underneath the canyon floor that surrounds each 5-fold axis of the viral capsid. Binding of specific inhibitors into this pocket increases virion rigidity, thus inhibiting attachment or disassembly of the viral particle after receptor binding (*10*). Consequently, the release of the viral genome into the host cell is prevented and viral replication is inhibited. Two of the most extensively characterized series of capsid-binding agents are the so-called WIN compounds, developed by Sterling Winthrop (New York, NY, USA), and a series of pyridazine analogs developed by the Janssen Research Foundation (Beerse, Belgium). The prototypes of these series are pleconaril (*11*) and pirodavir (*12*), respectively.

In clinical studies, pleconaril was a promising candidate for treating the common cold, but it was disapproved by the FDA in 2002, mainly because of possible interactions with other drugs, including those for birth control. Soon thereafter, pleconaril was licensed to Schering-Plough, which in 2007 completed a phase II clinical trial to study the effects of pleconaril nasal spray on common cold symptoms and asthma exacerbations following human rhinovirus (HRV) exposure. Meanwhile, pleconaril is still being used successfully on a compassionate basis for treating life-threatening enterovirus infections in children (*13*). Notably, it was effective in stopping virus excretion in a child persistently infected with PV, when combined with gamma globulin–mediated virus clearance (*14*). In another trial with a persistently infected person, however, treatment produced no benefit (*7*).

Intranasal pirodavir (R77975) was active in some clinical trials of human experimental rhinovirus infections, but lack of therapeutic efficacy and metabolic instability after oral administration halted further development. As shown in the Table, pleconaril and pirodavir, as well as a pirodavir analog (R78206) (*15*) inhibited PV2 and PV3 replication with EC_50_ values <2 μmol/L and TIs of 60 to >179. However, only R78206 exhibited inhibitory activity against PV1. Pirodavir proved 5- to 20-fold less active on PV1 replication, and pleconaril was inactive up to the highest concentration tested.

**Table:** Inhibitory activity of selected compounds against replication of poliovirus Sabin strains 1, 2, and 3 in HeLa cells, as determined by a CPE reduction assay*

Compound	EC_50_ (µmol/L)			Toxicity (CC_50_;µmol/L)	TI (min–max)
	PV1	PV2	PV3		
Capsid binders					
Pirodavir analog (R78206)	0.76 ± 0.18	0.22 ± 0.19	0.11 ± 0.10	27 ± 34	35–245
Pleconaril	>100	1.1 ± 0.6	0.22 ± 0.15	66 ± 6	<0.66–300
Pirodavir (R77975)	10 ± 1	1.7 ± 0.1	0.56 ± 0.03	>100	>10–>179
Protease inhibitors					
Rupintrivir	0.022 ± 0.028	0.041 ± 0.024	0.0052 ± 0.0046	>100	>2,439–>19,231
Compound 1	0.26 ± 0.24	0.31 ± 0 .21	0.060 ± 0 .000	>100	>322–>1,667
3A inhibitor					
Enviroxime	0.2 ± 0.25	0.056 ± 0.020	0.035 ± 0.029	58 ± 6	290–1,657
Nucleoside analogs					
Ribavirin	57 ± 13	64 ± 4	55 ± 7	>100	>1.6–>1.8
2'-*C*-methylcytidine†	15 ± 18	29 ± 27	3.9 ± 2.3	>100	>3.4–>26
2'-*C*-methyladenosine	5.5 ± 0.0	5.6 ± 0.1	5.4 ± 0.4	84 ± 0	15
4’-azidocytidine	>100	>100	>100	>100	><1
2C inhibitors					
HBB	300 ± 68	225 ± 128	295 ± 88	>400	>1.3–>1.8
MRL-1237	5.3 ± 0.3	4.6 ± 1.4	3.8 ± 2.5	>100	>19–>26
Unknown target					
MDL-860	6.0 ± 1.6	3.6 ± 2.2	2.2 ± 1.5	>100	>17–>45

*CPE, cytopathic effect; EC_50_, 50% effective concentration; CC_50_, 50% cytotoxic concentration; PV, poliovirus; TI, therapeutic index (CC_50_/EC_50_); min, minimum; max, maximum; HBB, 2-(α-hydroxybenzyl)-benzimidazole. †Valopicitabine (oral valine ester prodrug of 2′-*C*-methylcytidine).

### Protease Inhibitors

A second approach to inhibiting PV replication is by targeting the virus-encoded proteases 2A and/or 3C. These enzymes cleave the single polyprotein, encoded by the PV genome, into mature proteins. Rupintrivir (AG-7088, Pfizer, New York, NY, USA) is an irreversible inhibitor of the 3C function (*16*,*17*). Despite some successful trials in patients that were experimentally infected with HRV, rupintrivir was not able to mitigate disease severity in studies of natural rhinovirus infection, and clinical development was stopped (*18*). Further efforts by Pfizer resulted in the development of compound 1, an inhibitor with a similar mechanism of action and with an excellent oral bioavailability (*18*). Both compounds inhibited all 3 PV strains with EC_50_s <1 μmol/L and TIs of >322 to >19,230 (Table). Rupintrivir was the most potent compound of the selected series with EC_50_ values in the nanomolar range (5–40 nmol/L) against each of the 3 tested PV strains (Table).

### Protein 3A Inhibitors

Enviroxime is a benzimidazole derivative that inhibits the replication of enteroviruses and rhinoviruses in vitro by targeting the nonstructural protein 3A (*19*,*20*). Enviroxime inhibited the replication of all 3 strains of PV, with EC_50_ values of 35–200 nmol/L and TIs of 290–1,657 (Table). Previous in vivo studies with enviroxime, however, have shown toxicity and only weak to moderate activity, due to poor solubility and pharmacokinetics (*21*–*24*). Structural derivatives of enviroxime such as the C_2_- and vinylacetylene analogs were reported to have a better oral bioavailability and pharmacologic profile (*25*,*26*) and may therefore be considered as leading candidates for further development.

### Nucleoside Analogs

The nucleoside analog ribavirin is an antiviral drug with broad-spectrum activity against RNA and DNA viruses. Ribavirin is used in combination with interferon in the treatment of hepatitis C virus (HCV) infection (*27*) and as an aerosol to treat respiratory syncytial virus infections in children (*28*). As expected, ribavirin proved to be a weak inhibitor of PV replication with EC_50_ values of 50–60 μmol/L (TIs >1.6). Valopicitabine is the oral valine ester prodrug of another nucleoside analog, 2′-*C*-methylcytidine. The 5′-triphosphate of 2′-*C*-methylcytidine is an inhibitor of HCV polymerase (*29*). Clinical development of valopicitabine for the treatment of HCV infection was recently stopped, mainly because of gastrointestinal side effects. The compound was shown to exhibit relatively broad-spectrum activity against positive-sense single-stranded RNA viruses, including inhibition of the replication of foot-and-mouth-disease virus (*30*). It can be assumed that the mechanism by which 2′-*C*-methylcytidine inhibits picornaviruses is also by inhibition of the viral polymerase. As shown in the Table, 2′-*C*-methylcytidine inhibited the replication of PV strains with EC_50_ values of 3.9–29 μmol/L (TIs >3.4–>25.6). The adenosine analog of valopicitabine, as well as another nucleoside analog, 4′-azidocytidine (a potent inhibitor of HCV replication) were also included in this study. 2′-*C*-methyladenosine proved equipotent (≈5 μmol/L) against all 3 PV strains; whereas 4′-azidocytidine proved inactive (Table).

### Protein 2C Inhibitors

MRL-1237 and 2-(α-hydroxybenzyl)-benzimidazole (HBB) are inhibitors that target the enteroviral nonstructural protein 2C (*31*,*32*). MRL-1237 showed antiviral activity against PV strains 1, 2, and 3 with TIs >19. HBB appeared to be a weak inhibitor of PV replication with EC_50_s of 200–300 μmol/L and TIs >1.3.

### Compounds with Unknown Mechanism of Action

Compound MDL-860 was discovered as a broad-spectrum inhibitor of picornavirus replication, although the precise mechanism of antiviral activity has never been unraveled (*33*). The anti-PV activity of MDL-860 proved comparable to that of the 2C inhibitor MRL-1237.

**Figure 2:**
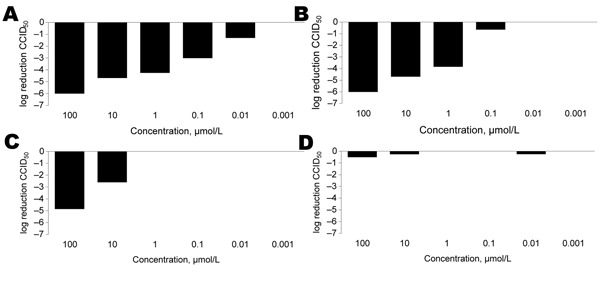
Effect of selected inhibitors on production of infectious poliovirus 1 Sabin in HeLa cell cultures. Supernatants collected from 3 independent experiments were titrated for infectious virus content, and 50% cell culture infective dose (CCID_50_) values were calculated as described by Reed and Muench (*34*). A) Ruprintrivir; B) enviroxime; C) MRL-1237; D) pleconaril.

To further confirm the activity observed in the CPE reduction assays, infectious virus yield reduction assays were carried out on the supernatant of PV1-infected cultures. As depicted in Figure 2, rupintrivir, the most active compound in the CPE reduction assay, caused a 6-log_10_ decrease of infectious virus production at 100 μmol/L, and reduced virus progeny formation 10–1,000-fold at concentrations of 10–100 nmol/L. Conversely, pleconaril, which did not protect against PV1-induced CPE formation, was not able to reduce infectious virus yield. A similar correlation between CPE formation and infectious virus production was observed for all other compounds included in the study (data not shown).

## Discussion and Perspectives

From our comparative study, rupintrivir and its analog compound 1 emerged as highly potent and broad-spectrum anti-PV compounds, without any signs of cytotoxicity up to the highest concentrations tested. The in vitro activity of these protease inhibitors against PV is comparable to their activity against various strains of HRV, the virus against which the compounds were originally developed (*17*,*35*). Given the excellent oral bioavailability (*35*) and its favorable pharmacokinetic profile, compound 1 may be an attractive candidate for further study for the treatment and prophylaxis of PV infection.

The pirodavir analog R78206 also displayed potent, broad-spectrum activity against PV. As was the case with rupintrivir, pirodavir did not appear to offer sufficient potential for treating HRV infection and was not further developed. The compound, however, was well tolerated; thus, pirodavir (and its analogs) may, alone or combined with other antiviral agents, open perspectives for treating PV infection. One major problem with pirodavir and its analog, however, is the poor pharmacokinetic profile after systemic dosing due to hydrolysis of the ester bond. Orally bioavailable analogs of pirodavir were developed at Biota (Melbourne, Victoria, Australia) but appeared to have a limited activity toward PV strains (*36*). Another compound that has undergone extensive clinical evaluation for enteroviral infections is pleconaril. The compound is relatively potent in inhibiting PV2 and PV3 replication, but has no activity against PV1, which limits its potential for PV. Besides pleconaril and pirodavir (analog), several other potent capsid-binding agents have been reported (reviewed in [*8*]).

Enviroxime, discovered in 1980, which exhibits potent anti-PV activity, was not developed because of unfavorable pharmacokinetics. However, a further exploration of the potential of enviroxime analogs could be worthwhile, in an attempt to improve the activity, selectivity, and in particular, the pharmacokinetic profile (*25*,*26*).

Compounds that have been less well characterized but that still may form a starting point for the synthesis of more potent and selective inhibitors of PV replication are MRL-1237 and MDL-860. Unraveling the precise mode of antiviral activity and the molecular interaction with their antiviral target may allow structure-based drug design.

Nucleoside polymerase inhibitors that have been developed for treating HCV infection may also have the potential to inhibit other single-stranded positive-sense RNA viruses. Here we demonstrate that the active component of the anti-HCV drug valopicitabine inhibits the replication of all 3 PV strains. If such a drug becomes available for treating HCV infections, it could also be used “off-label” to treat PV infection. However, 4′-azidocytidine, a potent inhibitor of HCV replication (*37*), was devoid of anti-PV activity up to the highest concentrations tested. As reported before and confirmed here, ribavirin proved to be a relatively weak inhibitor of PV replication (TIs >1.8). Although ribavirin has limited activity against HCV when used as monotherapy, its potency is markedly increased when it is given in combination with pegylated interferon. Since extensive clinical experience exists regarding the use of ribavirin in treating HCV infection, it may be possible and beneficial to explore the potential of the combined use of ribavirin with drugs such as rupintrivir, pirodavir, or their analogs.

Because of the high mutation rate of the viral RNA-dependent RNA polymerase, drug-resistant PV mutants have been readily selected in cell culture (*32*,*38*). The possibility that the use of antiviral drugs to treat polio would result in the appearance of drug-resistant variants cannot therefore be excluded. It should be noted, however, that the most potent inhibitors of in vitro PV replication that we identified here (the 3C inhibitors rupintrivir and compound 1, the capsid binders R78206 and pleconaril, and the 3A inhibitor enviroxime), act on different targets in the viral replication cycle. The use in combination of drugs with different modes of action will likely delay or prevent the emergence of drug-resistant variants. Moreover, the period of treatment during an acute PV outbreak would likely be much shorter than treatment regimens for such chronic infections as HIV or HCV, reducing the chance that drug-resistant strains will emerge.

As highlighted earlier, the need for adequate antiviral drugs against PV (most likely in combination with IPV) in the final stages of polio eradication is obvious. In a recent report from the World Health Organization (*39*), an advisory committee concurred with the proposal to establish a “PV antiviral initiative,” to take forward the key recommendations proposed during the National Research Council meeting on antiviral agents against PV.

In the present study, several drugs, some of which have been (rupintrivir, pirodavir, valopicitabine, compound 1) or are being (pleconaril) studied in the clinical setting, are reported to inhibit the in vitro replication of PVs to varying degrees. These drugs, used alone or in combination, may have potential for the treatment or prophylaxis of PV infections. These and other compounds may serve as starting points for the design of more potent PV inhibitors with favorable safety and pharmacokinetic profiles.
